# Analysis of Serum Cytokines and Single-Nucleotide Polymorphisms of SOD1, SOD2, and CAT in Erysipelas Patients

**DOI:** 10.1155/2017/2157247

**Published:** 2017-04-23

**Authors:** Charles C. Emene, Irina E. Kravchenko, Gulnaz I. Aibatova, Albert A. Rizvanov

**Affiliations:** ^1^Institute of Fundamental Medicine and Biology, Kazan Federal University, Kazan, Russia; ^2^Department of Infectious Diseases, Kazan State Medical University, Kazan, Russia

## Abstract

Increased free radical production had been documented in group A (*β*-hemolytic) streptococcus infection cases. Comparing 71 erysipelas patients to 55 age-matched healthy individuals, we sought for CAT, SOD1, and SOD2 single polymorphism mutation (SNPs) interactions with erysipelas' predisposition and serum cytokine levels in the acute and recovery phases of erysipelas infection. Whereas female patients had a higher predisposition to erysipelas, male patients were prone to having a facial localization of the infection. The presence of SOD1 G7958, SOD2 T2734, and CAT C262 alleles was linked to erysipelas' predisposition. T and C alleles of SOD2 T2734C individually were linked to patients with bullous and erythematous erysipelas, respectively. G and A alleles of SOD1 G7958A individually were associated with lower limbs and higher body part localizations of the infection, respectively. Serum levels of IL-1*β*, CCL11, IL-2R*α*, CXCL9, TRAIL, PDGF-BB, and CCL4 were associated with symptoms accompanying the infection, while IL-6, IL-9, IL-10, IL-13, IL-15, IL-17, G-CSF, and VEGF were associated with predisposition and recurrence of erysipelas. While variations of IL-1*β*, IL-7, IL-8, IL-17, CCL5, and HGF were associated with the SOD2 T2734C SNP, variations of PDFG-BB and CCL2 were associated with the CAT C262T SNP.

## 1. Introduction

Erysipelas is an acute bacterial infection of the upper part of the dermis and superficial lymphatic vessels. In addition to nonspecific symptoms of fever, chills, nausea, and vomiting, erysipelas has unique clinical features of infection including skin lesions, intense erythema, tenderness, and swelling of the lymph nodes. The legs and faces are the most common areas of localization for erysipelas [1, 2]. Group A (*β*-hemolytic) streptococcus (GAS) bacteria are the main causative agent for erysipelas that often began with breakages on the skin barriers allowing the microorganism to penetrate into the skin [3]. Diagnosis of erysipelas is largely based on clinical findings, but laboratory diagnosis of group A streptococcal infections is still largely used to identify the causative organism. GAS-related erysipelas has often been diagnosed in old and immunocompromised individuals as well as in neonates and young children [3]. The commonly accepted antibiotic for the treatment of erysipelas is penicillin, but it is important to note that since the 1980s, cases of severe invasive penicillin-resistant GAS diseases have been on the rise [4] and that to date, no licensed vaccines against GAS have been designed. A major clinical problem that has been encountered with erysipelas is its recurrence [3]. There are 18 million cases of severe GAS-related infections annually reported, with approximately 2.3% fatality [5]. It is believed that the severity of the disease and its fatality rate are associated with penicillin-resistant GAS [6]. Despite intense research, our knowledge of the GAS pathogenesis is incomplete. It has been shown that GAS-induced production of free radicals plays several roles in the pathogenesis of infections [1]. An increased production of free radicals can affect cell vitality by disrupting the integrity and function of cytoplasmic membranes, damaging membrane lipids, proteins, and nucleic acids. Additionally, free radicals can inhibit the immune system's ability to identify and eliminate GAS. These negative effects are referred to as oxidative stress and can be reduced with the aid of exogenous or endogenous antioxidants [2].

There are three main endogenous antioxidant systems: superoxide dismutase 1 (SOD1), superoxide dismutase 2 (SOD2), and catalase (CAT). SOD1 is found in the cytosol and nucleus, as well as in the intermembrane space of the mitochondria. SOD1 is a major cytoplasmic antioxidant enzyme that metabolizes superoxide radicals to molecular oxygen and hydrogen peroxide (Н_2_О_2_), thus protecting the cells against oxygen-related toxicity. SOD2 is the only antioxidant located exclusively in the mitochondria [7], where it scavenges the superoxide radicals and converts them to molecular oxygen and peroxide. Catalase (CAT) is generally found in the liver, kidney, and erythrocytes [5]. CAT catalyzes the hydrogen peroxide decomposition to harmless compounds such as water and oxygen. Therefore, antioxidants are important in preventing harmful effects of free radicals. Disturbed function of superoxide dismutase antioxidants can reduce cell protection against free radicals released during the course of infection [8].

Although cytokines play an important role in host defenses against infection, inflammatory cytokines may favor production of free radicals and facilitate oxidative stress. For example, overexpression of inflammatory cytokines TNF*α*, IL-6, IL-8, and IL-1*β* has been found in oxidative stress-induced melanomas [9]. Also, TNF-*α*-induced oxidative stress and subsequent redox system dysfunction have been described in patients with cardiac dysfunction [10]. In another study, the overexpression of IL-6 and TNF-*α* was shown to be associated with an increased severity of GAS infection, higher rate of toxic shock, and tissue necrosis [11, 12].

Currently, our knowledge on the association between SOD1, SOD2, and CAT gene expression and severity of erysipelas is limited. Relationships between serum cytokine levels and erysipelas infection are also scarcely known. Also, correlation between expression of SOD1, SOD2, and catalase genes and activation of inflammatory cytokine in erysipelas still remain unknown. Therefore, we sought to determine whether single-nucleotide polymorphisms (SNPs) in the genes encoding SOD1, SOD2, CAT, and cytokine levels and their interaction can serve as susceptibility markers for erysipelas. For that, we analyzed clinical characteristics of the disease in conjunction with SNPs in the selected genes and cytokine levels among residents of the Republic of Tatarstan, Russia. Additionally, relationships between the studied SNPs and serum cytokine profile were studied in erysipelas patients.

## 2. Materials and Methods

### 2.1. Subjects and Sample Collection

Seventy-one erysipelas subjects (average age 63.89 ± 10.33) admitted into the “Republican Clinical Infectious Diseases Hospital named after Professor A. F. Agafonova” of The Ministry of Health of the Republic of Tatarstan were enrolled into this study. Diagnosis was established based on clinical symptoms and laboratory bacterial culture test results. Subjects with concomitant severe and chronic inflammatory diseases in the acute phase were excluded. One blood sample was collected from each of the 71 erysipelas cases. Additionally, serum samples were collected from 50 patients, each of whom, one sample was taken from at the acute and another at the convalescent phase of the disease. The clinical characteristics of the subjects are summarized in Table 1. Fifty-five age-matched controls were recruited (average age 58.34 ± 6.93) as well. Blood samples were collected from 55 controls, while serum was obtained from 26 controls.

The Ethics Committee of Kazan State Medical University approved this study (N6, 06.25.2012), and informed consent was obtained from each study subject, in accordance with the Declaration of Helsinki and the Article 20, Federal Law “Protection of Health Right of Citizens of Russian Federation” (N323-ФЗ, 11.21.2011).

### 2.2. Genetic Analysis

Genomic DNA was extracted from the blood sample using phenol-chloroform protocol [13]. DNA was stored at −40°C until further use. DNA was analyzed for the presence of the SNPs in the SOD1 (G7958A; rs4998557), SOD2 (C60T, rs4880), SOD2 (T2734C; rs11575993), and CAT (C262T; rs101179) using SNP diagnostic kits 01327-100, 01333-100, 01281-100, and 01342-100, respectively (JSC “Lytech,” Russia). Amplification was performed according to the manufacturer's instructions.

### 2.3. Cytokine Analysis

A total of 44 cytokines were analyzed in the serum samples using Bio-Plex Pro™ Human Cytokine 27-plex and Bio-Plex Pro Human Cytokine 21-plex Assay multiplex kits according to the manufacturer's instructions. These cytokines include IL-1ra, IL-1*β*, IL-2, IL-2R*α*, IL-3, IL-4, IL-5, IL-6, IL-7, IL-8, IL-9, IL-10, IL-12 (p40), IL-12 (p70), IL-13, IL-15, IL-16, IL-17, IL-18, CCL2, CCL3, CCL4, CCL5, CCL7, CCL11, CCL27, CXCL1, CXCL9, CXCL10, CXCL12, FGF, G-CSF, GM-CSF, HGF, IFN-*α*2, IFN-*γ*, LIF, *β*-NGF, PDGF-BB, SCF, SCGF-*β*, TNF-*α*, TRAIL, and VEGF. A fifty-microliter serum sample was used to determine cytokine concentration according to the manufacturer's instructions. Data collected was analyzed using MasterPlex CT control software and MasterPlex QT analysis software (MiraiBio, Division of Hitachi Software, San Francisco, CA, USA).

### 2.4. Statistical Analysis

Statistical analysis of genotypes of the studied SNPs in our case-control study was carried out comparing the additive, dominant, and recessive models as earlier defined [14]. The Pearson *x*^2^ test, analysis of variance, and logistic regression were used to analyze the clinical differences between the subject groups and the control. Allele and genotype frequencies in the subject and control groups were compared with those predicted by the Hardy-Weinberg equations using *x*^2^ test. The Hardy-Weinberg equilibrium was satisfied in most of the distributions. The odds ratio (OR) and 95% confidence intervals (95% CI) were calculated to assess the association between the alleles and the risk of the studied infection. The association between cytokine levels, genotype, and clinical presentation of the study group was calculated using logistic regression and linear regressions using an earlier described web application SNPStats (http://bioinfo.iconcologia.net/SNPstats) [15]. Statistical difference (*p*) and Pearson's correlation coefficient (*K*) were used to analyze the cytokine profile of the patients. A value of *р* < 0.05 signifies a statistical significant difference in cytokine levels between groups and subgroups. The value of *K* > 0.4 was used to indicate a high degree of correlation in changes of a cytokine in infected individuals when the level of the cytokines during the acute phase is compared to the level of the same cytokine during the convalescent phase. Statistical analysis was performed using SPSS software 22 (IBM).

## 3. Results

### 3.1. Clinical Presentation

All erysipelas patients enrolled in this study were grouped based in the frequency of the infection: those without previous diagnosis of erysipelas (primary) within the last 365 days (38 patients) and others with more than one episode of erysipelas (recurrent) within the last 365 days (33 patients) (Table 1). Forty-eight patients were females, with an equal number of females having the primary form erysipelas (24 cases) and the recurrent form erysipelas (24 cases). Twenty-three were males, where 14 cases had primary and 9 had recurrent form of the infection. Erysipelas cases were analyzed based on the severity (moderate and severe) of the infection. A total of nine patients were diagnosed with the severe form of erysipelas, and 62 had a moderate form of the infection. The number of male and female patients with the moderate form of erysipelas was fairly similar (22 males; 95.7% of all males, versus 40 females; 83.3% of all females). However, a higher proportion of females had the severe form of the infection (8 females; 16.7%) than that of males (1 male; 4.3%). Based on the clinical manifestation of erysipelas, the subjects were also grouped based on the form of the infection into having erythematous form—33 patients (46.5%), and bullous form—38 patients (53.5%), of the disease. Erysipelas patients were also divided based on the localization of infection. The majority of patients were having infection on the lower limbs (83.1%), followed by the face (12.7%), and, least of all, on the upper limbs (4.2%). Additionally, a higher proportion of male patients had facial localization of the infection when compared to that of females (5 patients; 21.7% versus 4 patients; 8.3%). There were no significant differences between men and women having infection on the limbs.

From the observed results, we suggest that erysipelas has a higher chance to develop the severe form of erysipelas in females than in males. Also, more significantly higher number of female adults are prone to have the recurrent form of the infection than that of male adults but a higher proportion of males are prone to having erysipelas on their faces than that of females.

### 3.2. SNP's Predisposition to Erysipelas

#### 3.2.1. SOD1 G7958A

Association analysis revealed significantly higher predisposition to erysipelas in patients with G/G genotype (OR = 10.63, 95% CI = 4.45–25.40, *p* < 0.0001) as compared to patients with A/A or A/G genotype. When the G/G genotype was compared to combined G/A and A/A genotypes, the association with erysipelas was still significant (OR = 8.79, 95% CI = 3.76–20.56, *p* < 0.0001) in patients with G/G genotype (Table 2). These results suggest that individuals with the G/G genotype are predisposed to erysipelas as compared to other genotypes.

#### 3.2.2. SOD2 C2734T

An association was found between SOD2 C2734T SNP and the risk of erysipelas (Table 3). The T/T and C/T genotypes had a significant association with erysipelas. The T/T genotype had the highest predisposition to erysipelas (OR = 8.33, 95% CI = 2.44–28.41, *p* < 0.0007), followed by the C/T genotype (OR = 5.55, 95% CI = 1.98–15.55, *p* < 0.0011), and then the C/C genotype (OR = 1). It appears that the presence of a T allele (in T/T and C/T) increases the predisposition to erysipelas (OR = 6.19, 95% CI = 2.28–16.84, *p* < 0.0004) than that of C/C genotype. There was a lack of predisposition to erysipelas in SOD2 T/T genotype (OR = 2.3, 95% CI = 0.93–5.72, *p* < 0.0724) when compared to the C/T and C/C genotypes combined (Table 3). Our data suggest that individuals with the T allele (homozygous T/T or heterozygous C/T genotype) have a higher predisposition to erysipelas as compared to those with C/C genotype.

#### 3.2.3. SOD2 C60T

The frequency of the SOD2 C60T SNP allele and genotype distribution in the erysipelas patients and controls were not significantly different (Table 4). Therefore, we conclude that the SNP SOD2 C60T has a limited effect on the predisposition to erysipelas.

#### 3.2.4. CAT C262T

When analyzed individually, no association between genotypes C/C, C/T, and T/T and predisposition to erysipelas was found. However, when the predisposition was compared between patients with C/C genotype against those with T/T and C/T genotypes together, a significantly higher frequency of erysipelas was found in patients with C/C genotype (OR = 2.58, 95% CI = 1.24–5.38, *p* < 0.01) than in those with T/T and C/T genotypes combined (Table 5). Also, analysis revealed a lack of predisposition to erysipelas when patients with T/T genotype were compared to those with C/C and C/T genotypes. We conclude that the C/C genotype of CAT C262T may be linked to a higher predisposition to erysipelas than the C/T and T/T genotypes.

### 3.3. SNPs' Association with Clinical Characteristics of Erysipelas

The subjects were grouped based on gender, multiplicity of infection, lesion location, severity of the disease, and form of infection (Table 6). There were lack of differences in the distribution of SOD1 (G7958A), SOD2 (T2734C), SOD2 (C60T), and CAT (C262T) SNPs in subjects with erysipelas based on gender, multiplicity, and severity of the disease (*p* > 0.05). However, when SNPs were analyzed based on the form of erysipelas, a significant difference (*p* < 0.001) in frequency of SOD2 (T2734C) was found between the bullous and erythematous forms of erysipelas. There was a higher frequency of C allele in patients with erythematous erysipelas (48 patients—33.8%) as compared to that in patients with bullous form (37 patients—26.1%). In contrast, the frequency of patients with the erythematous form having the T allele was lower (18 patients—12.7%) than that in the patients with bullous form (39 patients—27.5%). Therefore, we concluded that the presence of C allele could define development of erythematous form of the disease, while T allele delineates bullous erysipelas. There were no differences in the distribution of the SOD1 (G7958A), SOD2 (C60T), and CAT (C262T) SNPs between patients diagnosed with erythematous and bullous forms.

Next, SNPs were analyzed based on the localization of erysipelas: the lower limbs, face, or the upper limbs (Table 6). We observed an association between SOD1 (G7958A) and localization of infection, where higher proportion of G/A and A/A genotypes was found in patients who had erysipelas on the upper limb, (*p* = 0.041). Conversely, a higher number of patients with the G/G genotype had the infection predominantly on the lower limbs. There was a lack of the association between the erysipelas location and SOD2 (T2734C), SOD2 (C60T), or CAT (C-262T) genotypes (Table 6).

### 3.4. Cytokine Status in Patients with Erysipelas

All cytokines analyzed in the erysipelas patients and control group were divided into four based on their expression levels during the acute phase and convalescent phase of the infection compared to their expression levels in the control group (Table 7).

Group 1 consists of IL-1*β*, CCL11, IL-2R*α*, HGF, CXCL9, and TRAIL. Serum level of these cytokines was significantly higher during the acute phase of the infection in the controls (*p* < 0.05). There was a lack of differences in these cytokine levels during the convalescent phase as compared to the controls (*p* > 0.05).

Group 2 included IL-7, IL-8, PDGF-BB, CCL4, and CXCL12 cytokines. Serum level of these cytokines did not differ in the acute phase of the disease when compared to the controls (*p* > 0.05); however, these cytokine levels differed significantly in the convalescent phase of erysipelas as compared to the controls (*p* < 0.05). The values of IL-7, IL-8, and PDGF-BB in patients were higher in the convalescent phase of the disease than those in controls. Interestingly, the serum level of CCL4 and CXCL12 was higher in the acute phase of the disease as compared to that in the convalescent phase.

Group 3 contained IL-5, IL-6, IL-9, IL-10, IL-13, IL-15, IL-17, G-CSF, GM-CSF, CCL2, CCL3, CXCL10, VEGF, MIF, SCF, and SCGF-*β* cytokines, which differed significantly in the acute phase and as well in the convalescent phase of the disease when compared to values in the control group (*p* < 0.05).

Finally, group 4 included IL-1ra, IL-2, IL-3, IL-4, IL12 (p40), IL-12 (p70), IL-16, IL-18, CCL5, CCL7, CCL27, CXCL1, FGF, IFN-*α*2, IFN-*γ*, TNF-*α*, TRAIL, and *β*-NGF. Serum level of these cytokines remained unchanged during the course of the disease and was similar to that in the controls (*p* > 0.05).

Since serum level of cytokines in groups 1 and 2 differed depending on the phase of the disease, we concluded that changes in these cytokines could be related to the disease pathology. Therefore, we focused our analysis on these cytokines. From cytokines in group 1, we found that the amount of reduction in values of IL1-*β*, CCL11, IL-2R*α*, CXCL9, and TRAIL from the acute phase to the convalescent phase was significantly similar in all the patients (*K* > 0.4). In group 2 cytokines, similar changes of serum levels of cytokines PDGF-BB and CCL4 from the acute phase to the convalescent phase were observed in all the patients (*K* > 0.4). Although the change was a similar reduction from the acute phase in CCL4, serum levels of PDGF-BB were on an average higher during the convalescent phase than the acute phase. Other cytokines of groups 1 and 2 did not express a similar change in values among the patients (*K* < 0.4). Therefore, we suggest that serum level of IL1-*β*, CCL11, IL-2R*α*, CXCL9, TRAIL, PDGF-BB, and CCL4 could serve as markers of clinical symptoms associated to erysipelas infection.

Since serum levels of group 3 cytokines in the patients differed from the values observed in the controls during the acute phase and also in the convalescent phase, we could suggest that these cytokines are associated to the predisposition of erysipelas and possible recurrence of the infection. Serum levels of IL-6, IL-9, IL-10, IL-13, IL-15, IL-17, G-CSF, and VEGFr expressed a similar change in values among the patients (*K* > 0.4) with the values of IL-6, IL-13, and G-CSF having a similar decrease in the patients from the acute phase to the convalescent phase; VEGFr is maintaining a similar level in the acute and convalescent phases, and IL-9, IL-10, IL-15, and IL-17 expressing a similar value increase from the acute phase to the convalescent phase. Other cytokines of group 3 did not express a similar change in value between the acute phase and the convalescent phase.

Cytokines in group 4 did not differ significantly in values between the patients and the controls, but a similar reduction in value from the acute phase to the convalescent phase in the patients was observed in FGF (*K* = 0.79), while a similar increase in value from the acute phase to the convalescent phase was observed in TNF-*α* (*K* = 0.83) and CCL27 (*K* = 0.43).

### 3.5. Correlation Analysis between Studied SNPs and Serum Cytokine Levels

Serum cytokines were analyzed in 50 erysipelas patients and 26 relatively healthy (control) individuals. Two serum samples were collected from each erysipelas case at the time of the admission (acute phase) and the time of discharge (convalescent phase). Erysipelas patients were grouped based on the identified SNPs in SOD T2734C and CAT C262T. Correlation between serum cytokines and SOD C60T was not analyzed since there was a lack of predisposition between this SNP and erysipelas. Also, the SOD 1 G7958A SNP correlation with serum cytokines was not analyzed due to a lack of control individuals with the A/A genotype.

#### 3.5.1. CAT C262T SNP and Serum Cytokines

The frequency of C/C and C/T genotypes within the 50 erysipelas patients was 25 (50%) and 25 (50%), respectively, while in controls, genotype distribution was 6 (25%) and 18 (75%), respectively.

Significant differences in the cytokine values when compared to the genotypes of CAT C262T were observed only in PDGF-BB and CCL2. Cytokine analysis revealed increased PDGF-BB level in serums of patients with C/T genotype (in the acute and convalescent phases) compared to that with controls (*p* = 0.045) (Table 8). Serum levels of CCL2 were significantly lower in the acute (*p* = 0.02) and the convalescent phases (*p* = 0.018) in patients with the CC genotype than in controls (Table 8).

#### 3.5.2. SOD2 T2734C SNP and Serum Cytokines

Distribution of C/C, C/T, and T/T alleles within the 50 erysipelas patients was 9 (18%), 38 (76%), and 3 (6%), respectively. The values observed in the 26 controls were 4 (15.4%), 17 (65.4%), and 5 (19.2%), respectively.

Although IL-1*β* level was elevated in the serum of erysipelas with T/T genotype as compared to the controls, differences in IL-1*β* levels were not significant between the acute and convalescent phases in the patients. There was a T allele dependency on IL-7, IL-8, IL-17, and CCL5 serum level in the patients during the acute phase of the infection. Interestingly, only HFG serum level was associated with the presence of C/C genotype in the erysipelas patients (Table 9).

## 4. Discussion

### 4.1. Demographic Distribution of Erysipelas

Erysipelas is a superficial skin infection with its etiology often linked to group A streptococci (GAS). The skin of the infected portion is often red, swollen, soft to the touch, and with clearly visible boundaries. Associated clinical symptoms may vary depending on the form of the infection, but typical symptoms could include fever, chills, malaise, and sometimes accompanied by nausea and vomiting [16]. A major problem of erysipelas to clinicians is its recurrence, which occurs in 8 to 23 percent of cases [17]. Erysipelas is mostly affected but restricted to the elderly; it has also been observed in younger people [18]. Our observed age distribution of the study group could be on one hand related to the fact that more elderly than younger people with erysipelas are prone to seeking medical care or that the elderly are at a greater risk [19]. Other research had also observed a similar distribution in the age of their studied group [18, 20]. Our study, which was restricted to people who could be categorized as elderly, showed that there was a sex dependence on the recurrence and severity of the infection with more elderly females having more of the recurrent and severe forms of the infection. The elderly female predisposition to recurrence could be associated to treatment or the presence of other ailments such as post breast cancer treatment [21], osteoporosis [22], and vaginal colonization of related microbes [23]. Gender-associated differences in the susceptibility to erysipelas could also be attributed to hormonal variations between male and female as has been previously shown by Bellanti et al. [24]. Our observation also revealed that a higher proportion of male patients had facial localization of the infection when compared to that of females.

### 4.2. SNPs' Association with Predisposition to Erysipelas

To date, our knowledge on SOD1 G7958A significance is limited to Yi et al. data presenting the association between the mutant allele (A) and increased expression of SOD1 mRNA, while the presence of the wild type (G) was related to reduced gene transcription and consequent increase and accumulation of free radicals in the cells [25]. Our data corroborates this observation, as a significant association was demonstrated between G allele of SOD1 G7958A and predisposition to erysipelas. We propose that the presence of G allele reduces transcription of SOD1 gene leading to decreased protein synthesis of the SOD1 enzyme. As a result, oxidative stress could develop leading to reduced antibacterial defense. Another finding showed that a dysfunctional or reduction in SOD1 expression favored the pathogenesis of H5N1 influenza virus [26] and amyotrophic lateral sclerosis [27, 28]. In another research, an in vitro model of amyotrophic lateral sclerosis showed that cells transfected with the mutant SOD1 were more vulnerable to infectious stimuli of the bacteria than cells overexpressing normal SOD1 [29]. We observed an association between SOD1 (G7958A) and localization of infection, where higher proportion of G/A and A/A genotypes were found in patients who had erysipelas on the upper limb. Conversely, a higher number of patients with the G/G genotype had the infection predominantly on the lower limbs. This differential distribution of the infection relative to SNP cannot be explained at the moment but needs to be studied.

The SOD2 C2734T polymorphism was shown to cause an amino acid substitution from leucine to phenylalanine at codon 84 of the SOD2 gene [30]. Our data provides evidence suggesting this SOD2 substitution of leucine to phenylalanine increases susceptibility to erysipelas and was linked to the more severe bullous form of the infection. It was proposed that due to this substitution, the produced protein subunits fail to properly interact, causing reduction of SOD2 activity and accumulation of free radicals in vitro [31]. Not much data is available about the role of the SOD2 C2734T, but an earlier research had showed that the SNP influenced susceptibility to vitiligo, a skin phenomenon characterized by long-term patches on the skin and loss of skin colour [32]. Other studies had shown that a dysfunctional SOD2 is positively associated with cardiovascular disease risk [33], neurodegeneration [34], diabetes [35], and different forms of cancers [36–38].

The C to T substitution in the 60th amino acid position of the SOD2 leads to an alanine to valine substitution. Our data demonstrated lack of association between the SOD2 C60T SNP and predisposition to erysipelas. Therefore, we suggest that SOD2 C60T SNP has limited role in pathogenesis of erysipelas.

The CAT C262T SNP is located in the promoter region regulating gene transcriptional activity. It has been shown that the C allele of CAT causes reduction of the transcriptional activity as compared to the T allele [9]. Additionally, C262C genotype of CAT was shown to produce more single-strand DNA breaks than C/T and T/T genotypes [9]. Our data demonstrate that erysipelas patients with the C allele have a higher risk of developing erysipelas. We believe that patients with C262C genotype have lower CAT activity, which can cause accumulation of free radicals and oxidative stress. The association between the CAT C262T SNP was found to have an effect on the predisposition to male infertility [39], *Shigella flexneri-*related infection [40], asbestosis [41], vitiligo [42], and cancer although it remains highly contradictory and inconclusive [43].

### 4.3. Cytokine Role in Erysipelas Pathogenesis

Our data suggest that IL1-*β*, IL-2R*α*, CCL4, CCL11, CXCL9, TRAIL, IL-1 *β*, and PDGF-BB cytokines could be linked to the clinical symptoms displayed during erysipelas infection. We have found that serum cytokine levels of these cytokines change during the course of the disease. The values of these cytokines during the acute phase in the patients significantly differed from that in the controls. However, during the convalescent phase, these cytokine levels returned to levels similar to that found in the controls. We also suggest that IL-6, IL-9, IL-10, IL-13, IL-15, IL-17, G-CSF, and VEGF could play a role in the clinical patterns and susceptibility of erysipelas infection. This suggestion was based on our finding that serum levels of cytokines in the acute and convalescent phases of the infection significantly differed from values observed in the controls. More research is however suggested to be able to draw out a more direct link between these cytokines and erysipelas infection.

### 4.4. Cytokine—SNPs' Interaction in Erysipelas

Activation of proinflammatory cytokines such as IL-1*β* is a hallmark of bacterial infections that is crucial for host-defense responses to infection and injury [10, 44]. The T allele of SOD2 C2734T was shown to be associated with decreased antioxidant capacity in the mitochondria and, subsequently, an increased oxidative stress. Our results show that increased serum IL1-*β* level in erysipelas patients is the T allele of SOD2 SNP C2734T dependent. Based on these observations, we propose that the T allele of SOD2 C2734T could trigger the upregulation of anti-inflammatory cytokines such as IL-1*β*, which would in turn attempt to stimulate the production of a functional SOD2 production which is downregulated in individuals with the T allele. This assumption is supported by our observation that serum IL-1*β* levels were higher in erysipelas patients homozygous for T allele than in patients with the heterozygous form. Therefore, we believe that serum levels of IL-1*β* could serve as a surrogate marker for predisposition to erysipelas and impaired antioxydase system. More study into the role of antioxydase system in erysipelas will help to understand the mechanisms of cytokine activation and their role in disease pathogenesis.

IL-7 regulates the development of T cells, enhances cytolytic T lymphocyte activity, and induces lymphokine-activated killer cells [45, 46]. Bacterial invasion induces IL-7 expression in T cells [47]. The role of IL-7 in oxidative stress remains largely unknown. However, studies suggest that IL-7 could be upregulated in patients with increased mitochondrial activity and oxidative stress [48, 49]. Our observation supports the notion that decreased mitochondrial activity is associated with upregulation IL-7. We have found upregulated serum IL-7 in patients with SNP in SOD2 suggesting decreased mitochondrial activity and increased ROS formation.

IL-8 is a chemoattractant for inflammatory leukocytes [50]. We found increased IL-8 in patients with the T allele of SOD2 C2734T that causes a decreased antioxidant activity. This observation supports previous observations which showed that IL-8 gene expression can be upregulated by free radicals, while antioxidants reduce the cytokine transcription [51]. We propose that infection could trigger oxidative stress and subsequently IL-8 production. These would exacerbate the generation of the reactive oxygen species and, when combined with decreased SOD2 activity, lead to perpetuate upregulation of IL-8.

The cytokine IL-17 is known to recruit leukocytes such as neutrophils to the site of infection thus aiding in the host's defense mechanism against extracellular bacteria. IL-17 also induces neutrophilic release of antimicrobial substances and ROS [52]. Our observation supports this observation as high IL-17 values were associated to the TT genotype of SOD2 C2734T and thus elevated oxidative stress.

CCL5 plays an active role in recruiting leukocytes and natural killer cells into inflammatory sites. A strong positive association between CCL5, ROS generation, and systemic lupus erythematosus had earlier been observed [53]. Our observation linked the high levels of expressed CCL5 in erysipelas patients who were linked to the homozygous T genotype in SOD2 C2734T SNP.

Interestingly, we have found increased serum level of HGF in erysipelas with C/C genotype of SOD2 C2734T. The C/C genotype is associated with the production of the functional SOD2 protein. Therefore, we suggest that expression of functional SOD2 protein, which is associated with properly functioning antioxidant, is linked to normal cell growth and differentiation. Our conclusion corroborates Borawski et al. data showing increased HGF in individuals with properly functioning SOD [54].

PDGF-BB is known for its participation in cell growth and differentiation. The mitogenic stimulus function of PDGF was shown to be mediated via ROS to activate cell proliferation [55]. Our research showed that while elevated values of PDGF-BB was observed in the erysipelas patients with the C/T genotype of CAT C262T during the acute phase and convalescent phase, the elevated value of PDGF-BB was observed in the patients with the C/C genotype only during the acute phase of the infection. As mentioned earlier, the C allele of CAT C262T was considered poorly functional and contributed to the predisposition of erysipelas. We propose that the functional T allele of CAT C262T contributed to maintaining high PDGF-BB values during the convalescent stage of the infection. Thus, the PDGF-BB-mediated T allele of CAT C262T aided in cell growth and differentiation to replace the erysipelas damaged cells.

CCL2 otherwise known as monocyte chemoattractant protein-1 (MCP-1) has been demonstrated to mediate the migration of monocytes from the blood stream across the vascular endothelium for routine immunological surveillance of tissues, as well as in response to inflammation [56]. The cytokine values of CCL2 were observed to be significantly lower in erysipelas patients with the C/C genotype of CAT C262T than in the individual in the control group with the C/C genotype. Values of the cytokine in patients with the C/T genotype were not significantly different from the values in the individuals of the control group with the C/T genotype. These observations lead us to propose that the C allele of CAT C262T which has been earlier shown to code for a poorly functioning catalase and higher predisposition to erysipelas is linked to low functioning of CCL2, thus hampering immunological surveillance of tissues in erysipelas patients.

## 5. Conclusion

Our research has been able to demonstrate that erysipelas infection predisposition and its clinical characteristics are affected by age, sex, and SNPs found in SOD1, SOD2, and catalase genes. These SNPs have an influence on an array of cytokines whose values were found to be different from values found in individuals not suffering from erysipelas.

## Figures and Tables

**Table 1:** Distribution of clinical characteristics of erysipelas in male and female patients.

		Male *n* = 23 (%)	Female *n* = 48 (%)	Total *n* = 71 (100.0%)
Multiplicity of infection	Primary	14 (60.9%)	24 (50.0%)	38 (53.5%)
	Recurrent	9 (39.1%)	24 (50.0%)	33 (46.5%)

Severity	Moderate	22 (95.7%)	40 (83.3%)	62 (87.3%)
	Severe	1 (4.3%)	8 (16.7%)	9 (12.7%)

Form	Erythematous	10 (43.5%)	23 (47.9%)	33 (46.5%)
	Erythematous-bullous	13 (56.5%)	25 (52.1%)	38 (53.5%)

Site of infection	Lower limbs	18 (78.3%)	41 (85.4%)	59 (83.1%)
	Face	5 (21.7%)	4 (8.3%)	9 (12.7%)
	Higher limbs	0 (0.0%)	3 (6.3%)	3 (4.2%)

Clinical characteristics of the patients were analyzed based on sex. Females have a higher chance of acquiring the infection and also severe forms than males, while a higher number of males have the infection on their faces than that of females.

**Table 2:** Distribution of SOD1 G7958A in control and erysipelas groups.

SOD 1 G7958A				
Genotype	Control	Erysipelas	OR (95% CI)	*p*
G/G	10 (18.9%)	47 (67.1%)	*10.63 (4.45–25.40)*	<0.0001
G/A	43 (81.1%)	19 (27.1%)	1	
A/A	0 (0%)	4 (5.7%)	NA	

G/G	10 (18.9%)	47 (67.1%)	*8.79 (3.76–20.56)*	<0.0001
G/A + A/A	43 (81.1%)	23 (32.9%)	1	

Patients with the G/G genotype had a higher frequency of the infection.

**Table 3:** Distribution of SOD2 SNP in the control and erysipelas groups.

SOD2 C2734T				
Genotype	Control	Erysipelas	OR (95% CI)	*p*
T/T	8 (14.6%)	20 (28.2%)	8.33 (2.44–28.41)	0.0007
C/T	27 (49.1%)	45 (63.4%)	5.55 (1.98–15.55)	0.0011
C/C	20 (36.4%)	6 (8.4%)	1	—

T/T + C/T	35 (63.6%)	65 (91.5%)	6.19 (2.28–16.84)	0.0004
C/C	20 (36.4%)	6 (8.4%)	1	

T/T	8 (14.6%)	20 (28.2%)	2.3 (0.93–5.72)	0.0724
C/T + C/C	47 (85.5%)	51 (71.8%)	1	

People with the T allele have a higher chance of developing the infection than people with the C allele.

**Table 4:** Distribution of SOD2 C60T SNP in the erysipelas and controls.

SOD2 C60T				
Genotype	Control	Erysipelas	OR (95% CI)	*p*
C/C	37 (67.3%)	57 (81.4%)	1.03 (0.16–6.44)	0.97
C/T	16 (29.1%)	10 (14.3%)	0.42 (0.06–2.94)	0.38
T/T	2 (3.6%)	3 (4.3%)	1	—

C/C + C/T	53 (96.4%)	67 (95.7%)	0.84 (0.13–5.23)	0.85
T/T	2 (3.6%)	3 (4.3%)	*1*	

C/T + T/T	18 (32.7%)	13 (18.6%)	*0.47 (0.21–1.07)*	0.07
C/C	37 (67.3%)	57 (81.4%)	*1*	

No significant association was observed between the SOD2 C60T and the infection.

**Table 5:** Distribution of CAT C262T SNP in the control and study groups.

CAT C262T				
Genotype	Control	Erysipelas	OR (95% CI)	*p*
C/C	18 (33.3%)	40 (56.3%)	6.67 (0.65–68.56)	0.11
C/T	33 (61.1%)	30 (42.2%)	*2.73 (0.27–27.66)*	0.40
T/T	3 (5.6%)	1 (1.4%)	1	—

T/T + C/T	36 (66.7%)	31 (43.7%)	1	0.01
C/C	18 (33.3%)	40 (56.3%)	*2.58 (1.24–5.38)*	

C/C + C/T	51 (94.4%)	70 (98.6%)	4.12 (0.42–40.73)	0.23
T/T	3 (5.6%)	1 (1.4%)	*1*	

The C/C genotype increases the predisposition to getting erysipelas infection.

**Table 6:** Association between SNPs and characteristics of patients and erysipelas symptoms.

		Gender (*n* (% genotype))		Multiplicity of infection (*n* (% genotype))		Lesion location (*n* (% genotype))			Severity (*n* (% genotype))		Form (*n* (% genotype))	
		Male	Female	Recurrent	Primary	Lower limbs	Face	Upper limbs	Low to mid	Severe	Erymanthus	Bullous
Genotype distribution												
SOD1 (G7958A)	G/G	13 (56.5)	34 (72.3)	23 (71.9)	24 (63.2)	42 (72.4)	5 (55.6)	0 (0.0)	41 (67.2)	6 (66.7)	18 (56.3)	29 (76.3)
	G/A	8 (34.8)	11 (23.4)	6 (18.8)	13 (34.2)	13 (22.4)	4 (44.4)	2 (66.7)	16 (26.2)	3 (33.3)	11 (34.4)	8 (21.1)
	A/A	2 (8.7)	2 (4.3)	3 (9.4)	1 (2.6)	3 (5.2)	0 (0.0)	1 (33.3)	4 (6.6)	0 (0.0)	3 (9.4)	1 (2.6)
*p*		0.406		0.203		0.041^∗^			0.538		0.165	

Allelic distribution												
	G	34	76	52	61	97	14	2	98	15	47	66
	A	12	15	12	15	19	4	3	24	3	17	10

Genotype distribution												
SOD2 (T2734C)	C/C	6 (26.1)	14 (29.2)	8 (24.2)	12 (31.6)	15 (25.4)	2 (22.2)	3 (100.0)	16 (25.8)	4 (44.4)	16 (48.5)	4 (10.5)
	C/T	14 (60.9)	31 (64.6)	22 (66.7)	23 (60.5)	39 (66.1)	6 (66.7)	0 (0.0)	40 (64.5)	5 (55.6)	16 (48.5)	29 (76.3)
	T/T	3 (13.0)	3 (6.3)	3 (9.1)	3 (7.9)	5 (8.5)	1 (11.1)	0 (0.0)	6 (9.7)	0 (0.0)	1 (3.0)	5 (13.2)
*p*		0.644		0.789		0.090			0.276		0.001^∗∗^	

Allelic distribution												
	C	26	59	38	47	69	10	6	72	13	48	37
	T	20	37	28	29	49	8	0	52	5	18	39

Only SOD1 (G7958A) and SOD2 (T2734C) with significant difference showed in some properties of the patients and their symptoms. Observed significance difference (*p* < 0.05) between the location of infection and SOD 1 (G7958A)^∗^. Significant difference was also found between the form of the infection and SOD2 (T2734C)^∗∗^.

**Table 7:** Cytokine profile of different phases of erysipelas infection.

	Binary logistic regression of cytokines comparing values in control to values in both phases of infection			95% confidence interval of cytokine values		Pearson's coeff (*K*) between both phases
	Phase	*p*	Average cytokine value	Lower	Higher	
Group 1						
IL-1*β*	Acute	0.046	1.516	1.008	2.280	^∗^0.436
	Convalescent	0.158	1.419	.873	2.308	
CCL11	Acute	0.002	.963	.941	.987	^∗^0.432
	Convalescent	0.962	.000	.000	7.555E + 216	
IL-2R*α*	Acute	0.009	1.072	1.017	1.130	^∗^0.495
	Convalescent	0.055	1.055	.999	1.115	
HGF	Acute	0.024	1.011	1.001	1.021	0.296
	Convalescent	0.099	1.010	.998	1.023	
CXCL9	Acute	0.016	1.015	1.003	1.028	^∗^0.436
	Convalescent	0.050	1.014	1.000	1.028	
TRAIL	Acute	0.049	1.053	.999	1.109	^∗^0.677
	Convalescent	0.091	1.043	.993	1.095	

Group 2						
IL-7	Acute	0.107	2.744	.805	9.357	0.340
	Convalescent	0.045	3.741	1.031	13.567	
IL-8	Acute	0.107	1.010	.998	1.023	0.340
	Convalescent	0.045	1.013	1.000	1.026	
PDGF-BB	Acute	0.072	1.010	.999	1.022	^∗^0.504
	Convalescent	0.044	1.016	1.000	1.031	
CCL4	Acute	0.052	1.579	.996	2.505	^∗^0.584
	Convalescent	0.044	1.016	1.000	1.031	
CXCL12	Acute	0.964	1.000	.988	1.013	−0.186
	Convalescent	0.031	.980	.961	.998	

Group 3						
IL-5	Acute	0.007	178.828	4.035	7925.340	0.001
	Convalescent	0.037	61.093	1.289	2896.100	
IL-6	Acute	0.007	1.053	1.014	1.094	^∗^0.500
	Convalescent	0.037	1.042	1.003	1.083	
IL-9	Acute	0.017	2.053	1.136	3.711	^∗^0.470
	Convalescent	0.033	2.641	1.082	6.449	
IL-10	Acute	0.017	1.075	1.013	1.140	^∗^0.470
	Convalescent	0.033	1.102	1.008	1.205	
IL-13	Acute	0.013	1.092	1.019	1.171	^∗^0.574
	Convalescent	0.012	1.091	1.019	1.169	
IL-15	Acute	0.006	1.119	1.034	1.211	^∗^0.420
	Convalescent	0.001	1.157	1.058	1.265	
IL-17	Acute	0.018	1.094	1.015	1.178	^∗^0.442
	Convalescent	0.020	1.106	1.016	1.204	
G-CSF	Acute	0.019	1.153	1.024	1.297	^∗^0.607
	Convalescent	0.015	1.134	1.025	1.255	
GM-CSF	Acute	0.014	1.014	1.003	1.025	−0.167
	Convalescent	0.031	1.011	1.001	1.021	
CXCL10	Acute	0.011	1.005	1.001	1.009	0.296
	Convalescent	0.040	1.004	1.000	1.008	
CCL2	Acute	0.035	1.225	1.014	1.481	0.171
	Convalescent	0.010	1.337	1.071	1.671	
CCL3	Acute	0.035	675657538.718	4.009	113865012433100720.000	
	Convalescent	0.010	4221003831075.196	914.082	19491539205039153000000.000	0.171
VEGFr	Acute	0.019	1.036	1.006	1.067	0.767
	Convalescent	0.040	1.036	1.002	1.071	
MIF	Acute	0.027	1.017	1.002	1.032	0.360
	Convalescent	0.019	1.021	1.003	1.039	
SCF	Acute	0.020	1.058	1.009	1.110	0.386
	Convalescent	0.006	1.124	1.034	1.222	
SCGF-*β*	Acute	0.039	1.003	1.000	1.006	0.293
	Convalescent	0.021	1.003	1.000	1.006	

Group 4						
IL-1ra	Acute	0.323	1.022	.979	1.067	0.363
	Convalescent	0.549	1.015	.966	1.068	
IL-2	Acute	0.085	1.679	.930	3.030	0.108
	Convalescent	0.068	1.677	.963	2.922	
IL-4	Acute	0.093	2.670	.850	8.392	0.131
	Convalescent	0.380	1.618	.552	4.743	
IL12 (p70)	Acute	0.093	1.035	.994	1.077	0.237
	Convalescent	0.634	.989	.945	1.035	
FGF	Acute	0.116	1.010	.998	1.022	^∗^0.792
	Convalescent	0.421	1.005	.993	1.017	
IFN-*γ*	Acute	0.093	1.003	.999	1.007	−0.010
	Convalescent	0.764	1.001	.997	1.005	
CCL5	Acute	0.106	1.004	.999	1.009	−0.235
	Convalescent	0.059	1.007	1.000	1.015	
TNF-*α*	Acute	0.325	1.030	.971	1.092	^∗^0.825
	Convalescent	0.092	1.123	.981	1.284	
IL-3	Acute	0.086	1.020	.997	1.043	0.179
	Convalescent	0.647	1.005	.984	1.027	
IL12 (p40)	Acute	0.650	1.002	.993	1.012	0.244
	Convalescent	0.606	.998	.988	1.007	
IL-16	Acute	0.323	1.010	.990	1.031	−0.205
	Convalescent	0.688	.996	.975	1.017	
IL-18	Acute	0.697	1.013	.950	1.080	0.163
	Convalescent	0.556	1.020	.956	1.088	
CCL27	Acute	0.299	1.009	.992	1.027	^∗^0.428
	Convalescent	0.209	1.015	.992	1.038	
CXCL1	Acute	0.108	1.162	.968	1.397	0.005
	Convalescent	0.252	1.167	.896	1.521	
IFN-*α*2	Acute	0.157	1.092	.967	1.233	0.264
	Convalescent	0.502	1.042	.924	1.174	
CCL7	Acute	0.269	.983	.953	1.014	0.230
	Convalescent	0.120	.967	.927	1.009	
*β*-NGF	Acute	0.056	7.255	.949	55.459	0.071
	Convalescent	0.489	2.543	.181	35.767	

Column *p* indicates the statistical difference of cytokine values between the respective phases and the cytokine values found in the controls. (Group 1) cytokine values in studied patients in the acute phase of the infection significantly differed from values observed in the controls (*p* < 0.05). During the convalescent stage, the values of the cytokines recovered to levels observed in the healthy patients (*p* > 0.05). ^∗^Coefficient of changes in cytokine values, except in HGF, of the patients between the two phases was high (*K* > 0.04). (Group 2) cytokine values in the studied patients did not differ from values observed in the healthy participants (*p* > 0.05) but differed from the values in the controls during the convalescent stage (*p* < 0.05). ^∗^Coefficient of changes in cytokine values of PDGF-BB and CCL4 of the patients between the two phases was high (*K* > 0.04). (Group 3) cytokine levels in the acute and convalescent phases significantly differed from the values of these cytokines in healthy participants (*p* < 0.05). Similar changes between both phases in the patients were observed in IL-6, IL-9, IL-10, IL-13, IL-15, IL17, and G-CSF (*K* > 0.04). (Group 4) these include cytokines which had no statistically significant difference in values between both stages in the values of the studied patients and the healthy participants (*p* > 0.05). However, FGF, TNF*α*, and CCL27 had a similar change in level in the patients.

**Table 8:** Cytokine concentration respective on genotypes of CAT C262T.

Cytokine mean value *μ*g/ml of serum (standard error)				
Cytokines	Genotype	Control	Acute phase	Recovery phase
PDGF-BB	C/C	43.22 (15.26)	122.84 (62.44)	79.32 (34.93)
	C/T	43.35 (12.68)	252.26 (61.5)	253.61 (53.45)

CCL2	C/C	42.81 (25.15)	5.71 (3.21)	1.34 (0.1)
	C/T	13.08 (8.1)	16.34 (6.24)	10.44 (4.26)

The table displays cytokines with significant differences in mean values between the control values and/or the acute phase and convalescent phase. The *p* values were not included in the table.

**Table 9:** Cytokine concentration respective on genotypes of SOD2 (T2734C).

Cytokine (*μ*g/ml) mean value (standard error)				
Cytokines	Genotype	Control	Acute phase	Recovery phase
IL-1*β*	C/C	4.3 (2.4)	6.43 (1.7)	3.82 (1.28)
	C/T	3.14 (0.35)	4.96 (0.7)	4 (0.43)
	T/T	3.03 (0.29)	12.2 (2.1)	11 (1.94)

IL-7	C/C	0.7 (0.29)	1.25 (0.5)	1.78 (0.63)
	C/T	0.82 (0.18)	1.81 (0.46)	1.29 (0.26)
	T/T	0.46 (0.09)	8.71 (0.31)	1.55 (0.27)

IL-8	C/C	69.5 (29.5)	124.67 (49.59)	178 (62.77)
	C/T	82.5 (18.27)	180.76 (45.65)	129.07 (25.9)
	T/T	46.33 (9.13)	871 (36.96)	155.05 (27.42)

IL-17	C/C	19.8 (11.3)	28.57 (8.58)	21.93 (3.64)
	C/T	16.32 (3.79)	30.72 (3.89)	26.59 (2.57)
	T/T	17.03 (2.51)	103.2 (23.37)	50.3 (3.62)

CCL5	C/C	291.9 (11.8)	669.7 (253.4)	656.97 (146.29)
	C/T	303.23 (44.96)	627.41 (184.25)	438.27 (66.07)
	T/T	232.13 (19.79)	15620.2 (7505.73)	198.1 (52.73)

HGF	C/C	69.95 (54.15)	709.16 (333.39)	86.23 (19.14)
	C/T	68.56 (18.55)	188.71 (40.41)	136.95 (30.75)
	T/T	45.48 (38.55)	28.36 (7.32)	20.7 (6.26)

The table displays cytokines with significant differences in mean values between the control values and/or the acute phase and convalescent phase.
